# New Insights into the Management of an EHV-1 (Equine Hospital) Outbreak

**DOI:** 10.3390/v13081429

**Published:** 2021-07-22

**Authors:** Eveline Vandenberghe, Berit Boshuizen, Catherine J. G. Delesalle, Lutz S. Goehring, Katy A. Groome, Kees van Maanen, Cornelis M. de Bruijn

**Affiliations:** 1 Hagyard Equine Medical Institute, 4250 Iron Works Pike, Lexington, KY 40511, USA; 2 Wolvega Equine Hospital, Stellingenweg 10, 8474EA Oldeholtpade, The Netherlands; bboshuizen@dierenkliniekwolvega.nl; 3 Research Group of Comparative Physiology, Department of Virology, Parasitology and Immunology, Faculty Medicine, Ghent University, 9820 Merelbeke, Belgium; Catherine.delesalle@ugent.be; 4 Equine Hospital, Center for Clinical Veterinary Medicine, Division of Medicine and Reproduction, LudwigMaximilians University, 80539 Munich, Germany; Goehring@pferd.vetmed.uni-muenchen.de; 5 The Royal Veterinary College, Royal College Street, London NW1 0TU, UK; kgroome@rvc.ac.uk; 6 GD Animal Health, Department of Small Ruminants, Horses and Companion Animals, Arnsbergstraat 7, 7418EZ Deventer, The Netherlands; c.v.maanen@gddiergezondheid.nl

**Keywords:** equine herpes myeloencephalopathy, quarantine, vaccination

## Abstract

In May 2018, Wolvega Equine Hospital (WEH) experienced an EHV-1 outbreak. This outbreak caused significant economic losses and negative publicity for the hospital. How should hospitals prepare themselves for these outbreaks and prevent shedding of the virus on multiple neighboring premises? The hospital transformed most of its activities into mobile practice and the entire infected hospital population was moved to a separate remote location. The hospital was cleaned and disinfected according to the latest recommendations before reopening. Four neighboring professional equine businesses and three privately owned premises were affected by the spread of the virus from the hospital population and initiated quarantine restrictions. Equine hospitals should prepare themselves for EHV-1 outbreaks as the intake of the virus cannot be prevented. A management protocol should include public information protocols, swift client information and quarantine measures that ensure quick containment of the outbreak. Timely reopening of the hospital can be achieved by rehousing the contaminated population. It should also include good regulations with clients and a properly carried out release protocol. Equine sports organizations should establish sufficient vaccination coverage in order to decrease the frequency of EHV-1 outbreaks.

## 1. Introduction

Equid herpesvirus 1 (EHV-1)-associated myeloencephalopathy (EHM) is a highly complex and multifactorial equine disease. It is considered a complication following EHV-1 respiratory tract infection and viremia. EHV-1 is highly contagious with the potential to cause an outbreak on multiple neighboring premises and have major consequences for the Equidae affected, including permanent neurological deficits or even death [1]. Alongside this lies a huge economic impact for the equine industry concerned.

The primary infection with EHV-1 takes place in the upper airway epithelium. After inhalation, the virus enters the cells of the respiratory tract where it replicates. From here, the virus spreads systemically, causing a primary viremia. A secondary phase of infection can then ensue, this is termed “leucocyte-assisted viremia”, and leads to dissemination of the virus to several organ systems, one of which is the central nervous system (CNS). The summative effects of this multi-focal disease throughout the CNS cause neurological gait anomalies, ataxia, dysmetria, paresis and, if severe enough, (tetra)paralysis. EHV-1 infection results in a chronic–persistent infection or latency in the lymphoreticular system and the trigeminal ganglion, from which the virus may later be reactivated in response to still poorly defined stimuli [2,3]. Fever during primary infection is absent, mild or moderate, and after two to four days, there is a normalization in temperature, which may be followed again by a sudden increase in temperature (up to 40 degrees Celsius) for a further three to seven days [4]. Nasal shedding starts with the primary infection through aerosolization of nasal fluids when infected cells in the respiratory tract burst open [2,3]. This horizontal spread is usually highest for the first five days; however, this can stretch out over the period of viremia, 1 to 3 weeks post infection, and sometimes past the point when EHM has already developed [2].

There are a few diagnostic tests ante mortem. The gold standard for detecting EHV-1 is virus isolation, although the most practical way is by quantitative polymerase chain reaction (qPCR) of nasopharyngeal swabs and/or venous blood collected in EDTA during viremia. The test has a high sensitivity and specificity, is cost effective and supplies results quickly [2]. Another benefit of qPCR is that it can also differentiate between viral shedding via nasopharyngeal secretions in clinically affected/infected horses and subclinically infected horses, as this group is not actively shedding the virus in nasal secretions and therefor will test negative on qPCR [5]. Follow up assessment is indicated to guide the modifications to quarantine restrictions during an outbreak, including when such measures are to be declared free after repeated negative sampling. Another diagnostic test is seroconversion, requiring an initial sample, and then a second sample collection 7 to 21 days later, which should demonstrate a fourfold titer increase to denote a horse’s positive infection. A limitation to this test is the need for the first sample, which is not always obtained. A third ante mortem test for EHM is analysis of cerebrospinal fluid (CSF) with characteristic fluid changes, such as xanthochromia appearance, an increased protein concentration and an increased albumin quotient [2]. These characteristic fluid changes are not pathognomonic for EHV-1, as other diseases such as septic meningitis can give a similar result. This test is more technically difficult to perform, as fluid needs to be obtained from the C1–C2 region or lumbosacral region, which can be difficult in highly muscled horses. The safety concerns when performing a spinal tap procedure on an ataxic horse with a risk of reaction during the procedure make it more difficult. Despite these limitations, this test can be supportive in confirming a diagnosis while qPCR results are pending [2,6].

It is widely believed that there has been a recent increase in the prevalence of EHV-1 outbreaks [6]. During an outbreak, the majority of a herd will develop a primary infection with 10–40% going on to develop EHM. This is partially due to strain differences within EHV-1; the D_752_ genotype is more often associated with neurologic outbreaks of EHV-1, while the N_752_ genotype is more often associated with non-neurologic outbreaks [2]. It is suggested that the majority of the circulating EHV-1 strains consist of the N_752_ genotype and it seems that horses exposed to the D_752_ genotype can be latently infected even if they were already latently infected with the N_752_ genotype [7]. However, several EHM outbreaks have been described as being caused by the N_752_ genotype, therefore it is unclear if the D_752_ genotype is a strong virulence marker. Development of viremia is again influenced by strain differences, with D_752_ inducing a robust viremia in duration and magnitude and increased likelihood of EHM [8]. Individuals presenting with high fever several days after the initial onset of fever are more likely to develop EHM [9]. Other factors influencing the onset of infection are the viral dose, the local immunity in the respiratory tract and the biosecurity in the case of inappropriate biosecurity measures. Other risk factors for EHM that have been discussed include immunity, age, breed, season and the duration of viremia [2,3,10]. The virus is highly contagious and there are various components that play a role in the spread of EHV-1. EHV-1 is usually transmitted by direct (nose-to-nose) contact, through aerosols, which may have played a significant role during the high case fatality rate in the Valencia 2021 outbreak. Furthermore, in abortion cases caused by EHV-1, virus can be found in fetal fluids and membranes as a result of high viremic loads [6]. Horses with signs of EHM also likely still shed the virus via the respiratory tract. While it is still unclear how long the duration of nasal shedding of EHV-1 can be expected to last [11], some can shed up to 4–8 weeks after the onset of neurological deficits [12]. It is therefore essential to have reliable preventative measures in place, whilst also having a clear protocol to follow in the case of an outbreak, from an individual through to national outbreak perspective. The introduction of a latently infected horse to an equine hospital, stud, training stable, livery yard or any other high-traffic equine facility is unavoidable, with the probability of viral shedding being even higher in an equine hospital, where there can be a reactivation of the virus due to stress, immunosuppression or the original problem.

**Figure 1:**
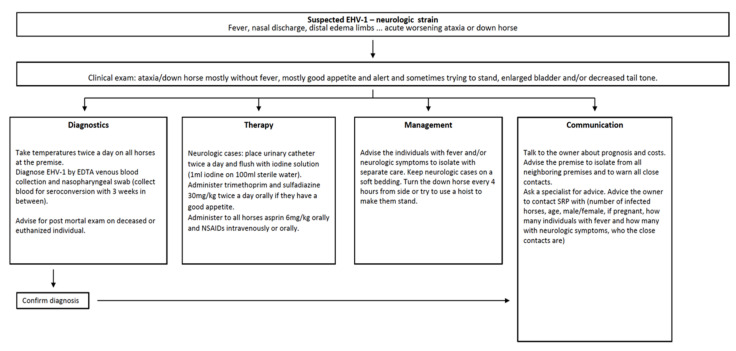
Diagnosis, treatment and management of suspected EHV-1 infection. Adapted from [13] with permission from Koninklijke Nederlandse Maatschappij voor Diergeneeskunde, Copyright 2014.

In the Netherlands, there is a guideline provided by the Koninklijke Nederlandse Maatschappij voor Diergeneeskunde (Royal Dutch Society for Veterinary Medicine-KNMvD) for equine herpes virus called “Rhinopneumonie bij het paard” (“Rhinopneumonia in the horse”) [13]. These guidelines provide veterinarians with information on how to give advice to horse owners during an outbreak situation. It is presented in Figure 1 in flowchart form, with information on diagnostics, treatment and prevention of the different clinical syndromes (abortion, respiratory and neurological diseases) caused by EHV-1 [13]. The flowchart is readily interpretable, however, it fails to provide clear instruction on “what to do in the event of an outbreak”, from the first moment of suspicion until resolution of the outbreak. The KNMvD gives recommendations on how to report outbreaks and who to report to, including directly to the Sectorraad Paarden (SRP, Board of Equine Businesses). Sectorraad Paarden subsequently collects all reports of cases/outbreaks, and publishes them on the SRP website. The KNHS stands to oversee and regulate equestrian sport in the Netherlands. In 2019, the KNMvD and Gezondheidsdienst voor Dieren (Animal Health Service—GD) together developed a surveillance system for EHV-1, EHV-4, strangles and equine influenza called “Surveillance Equine Infectieziekten Nederland” (Dutch Surveillance of Equine Infectious Diseases—SEIN), however, it is still not mandatory to report cases of EHV-1.

A major factor influencing the enforcement of compulsory reporting of cases to the relevant authorities is the negative publicity (for example, on social media) the affected premises could potentially receive. Not knowing which premises are infected, however, results in increased risk for a widespread epidemic, due to a lack of preventative measures being undertaken. In 2014, Merck Sharp and Dohme (MSD), a pharmaceutical company, undertook research into vaccination rates by horse owners. Results revealed a worryingly low vaccination rate, with only 15% of the registered equine population in the Netherlands vaccinated for EHV-1 and 22% vaccinated for EHV-1 and EHV-4 [14], resulting in a population that remains vulnerable to infection.

In this article, we will focus on how to manage an EHV-1 outbreak in a hospital setting, the clinical outcomes following infection, disease transmission and an effective quarantine protocol. We will also discuss how to deal with repercussions of infection, such as bad publicity (including on social media), and communication with both the equine businesses concerned and the public.

## 2. Materials and Methods

### 2.1. History of the Equine Hospital Outbreak

In January 2018, Wolvega Equine Hospital (WEH) admitted a horse with a fever of unknown origin. The horse had tested positive for EHV-1 and strangles two days before the consultation at the clinic. These test results were not known on arrival. The horse had participated in a CHIO competition in the local area. It is possible that this particular horse (and maybe others) had been shedding the virus and infected several horses at the same competition. Most of these horses were residing in the northern part of the Netherlands. On 26 April, one of the ambulatory veterinarians at the hospital was called to an emergency at “Premise A”; the horse was ataxic, which then progressed to dog sitting and complete tetraplegia within twelve hours. The horse had been hospitalized for two days at the hospital for a lameness examination. A similar case appeared later, albeit with milder neurological symptoms, on 28 April at “Premise D”; the affected horse had been hospitalized for eight days for insemination with frozen semen. This means that both horses had been hospitalized at the equine hospital before neurological symptoms appeared. On 28 April, two hospital-owned teaser mares became pyrexic. One of the mares became ataxic and, by 2 May, was tetraplegic. Test results from samples taken on 30 April tested positive for EHV-1 via PCR on nasopharyngeal swabs and EDTA blood. The complete hospital population was subsequently put under quarantine measures according to KNMvD guidelines. As of 2 May, there were a total of 40 in-patients throughout the different departments of the hospital.

Appointments and surgeries for the following 14 days were canceled. The owners of the hospitalized horses that had been discharged in the 10 days prior to 28 April were notified; three of these later developed a fever, with two testing positive for EHV-1, and quarantine protocols were also initiated at the affected premises.

At the hospital, extra biosecurity measures were put in place for the individuals with a fever and/or neurological symptoms, as suggested by the KNMvD [14]. The hospital population was divided into 3 groups: barn Q (EHV-1 positive), barn B (in contact/pyrexic) and barn Z (no symptoms). Between barns, staff had to change clothes and gloves, and equipment such as twitches and muzzles were disinfected to minimize fomite transmission after each use. Despite these measures, the EHV-1 spread and more than 50% of horses became pyrexic within four days, with six of these 40 horses (15%) developing neurological symptoms. Most likely, the virus had spread during the incubation period via staff, attending veterinarians and aerosolization directly between horses within each barn.

In total, there were four neighboring premises and three individually owned horses that reported positive EHV-1 samples and subsequently initiated quarantine restrictions. Premises A, B, C and D (commercial properties) and E, F and G (private properties) had horses hospitalized before 2 May, meaning there were 8 possible sources of origin within a range of 50 km. This means that the transmission between WEH and premises occurred via discharged patients before quarantine measurements were applied. Private property E had a mare and her foal hospitalized; all others just had one horse hospitalized. This helped to explain the scale of the outbreak, with details on how the premises were connected to each other, and that the transmission occurred via direct contact, rather than by aerosol. The neighboring riding schools, within a range of 10 km, canceled all lessons, although they were not officially obligated. Each operation isolated the EHV-1 positive horses, and all those who had been in contact were monitored. These premises reopened only when all horses had one negative test result three weeks after the last day of fever. Table 1 represents how long each possible horse of origin had been hospitalized, and how many days before closure of the hospital they had been discharged.

### 2.2. Quarantine on Location

The hospital (in-house) and ambulatory veterinarians were completely separated. The ambulatory veterinarians were not allowed to enter the hospital past reception. Stocking of their vehicles was organized. Outpatient appointments were moved to another location 800 m further down the road. The hospital transformed most of its activities into an ambulatory practice. The hospital remained a “restricted area” for ten days for visitors, owners and ambulatory hospital staff. A team of 4 people handled the hospitalized animals. The complete hospital population was eventually moved to a separate remote location, a stable unit 800 m away from the hospital, on 11 May. Three teams (A–C) were established: (A) stayed at the hospital to attend, (B) transported the horses between the 2 sites and (C) stayed in the new quarantine zone. The team based in quarantine was not allowed into the hospital under any circumstances, until all their horses had been released. A “drop-off site” was established for the delivery of medical supplies to ensure no members of the “clean team” became contaminated.

After moving the hospital population to the new quarantine facility, all employees (with the exception of the quarantine team) helped to clean and disinfect (chlorine) the hospital over a 48 h period. After two days of cleaning followed by two days of drying, the hospital reopened for regular business. EHV-1 is purportedly environmentally labile and does not survive well outside its equine host; it should, therefore, be easily killed by treatment with detergents, lipid solvents, heat and common disinfectants. Saklou at al., however, showed that EHV-1 can survive 48 h on environmental material [15]. Experimentally, the virus has survived for up to a week at ambient temperatures when dried into paper, wood and rope and up to 35 days on horsehair and burlap [16]. Therefore, despite its described fragile nature, EHV-1 could remain infectious on selected fomites under suitable conditions for up to a month (although the typical survival time would probably be much shorter) [17].

### 2.3. Treatment of Individuals and Follow-Up

The patients in the quarantine zone were treated as described in the guidelines of the KNMvD [13]. The hospital provided the appropriate supportive care, such as good nutrition and hydration, rectal examination and, if necessary, evacuation of retained manure and urine (via catheterization). Medical treatment consisted of enteral meloxicam (0.6 mg/kg) (Metacam; Boehinger Ingelheim, Germany) to reduce CNS inflammation, and prophylactic treatment with trimethoprim–sulfamethoxazole (5 mg/kg trimethoprim and 25 mg/kg sulfadiazine) (Sulfatrim, Wilgenweg, The Netherlands) in cases where urinary catheterization was necessary. As soon as neurological symptoms appeared, the patients were administered dexamethasone (0.06 mg/kg) (Dexa-Ject, Zalmweg, The Netherlands) once to decrease inflammation. No antiviral or anti-thrombotic therapy was administered.

The quarantine population had nasopharyngeal swabs taken for the first time on 7 May to identify the EHV-1-positive individuals. The second samples were taken 21 days later on 29 May, and horses were released when the first tested sample of each patient was negative three weeks after the last day of reported fever. The third and fourth rounds of tests were on 7 June and 13 June, respectively, to identify which individuals had become negative and presumably were no longer shedding the virus.

### 2.4. Communication

The hospital informed the Dutch board of equine industry (“Sectorraad Paarden”) as soon as the first horse was confirmed as EHV-1 positive on 2 May. There was frequent communication with the Animal Health Service (Gezondheidsdienst—GD) and the faculty of Veterinary Medicine at Utrecht University, for both updates on the situation and advice. The hospital provided situation reports on a regular basis (every few days) via its website and Facebook page. Interviews were published in the local and regional newspapers, and broadcast from the regional radio station. An informative meeting was held for around 500 members of the public, with up-to-date information regarding EHV-1 pathophysiology, transmission, preventative measures and a briefing on the current status of the outbreak. At the hospital, a provisional help desk was installed where all enquiries via telephone and email were processed, categorized and answered by a specialist in internal medicine. The owners of the quarantined hospital population were informed daily on the status of their horses.

Affected clients only had to pay for the costs of the original reason of admission to the hospital, and no charges were applied for the subsequent hospitalization, diagnostics and treatment due to the EHV-1 outbreak. The owners of affected premises were offered support in every possible way; however, they were not compensated for expenses or services supplied by other veterinary practices.

## 3. Results

### 3.1. Outcome Hospital Population

**Figure 2:**
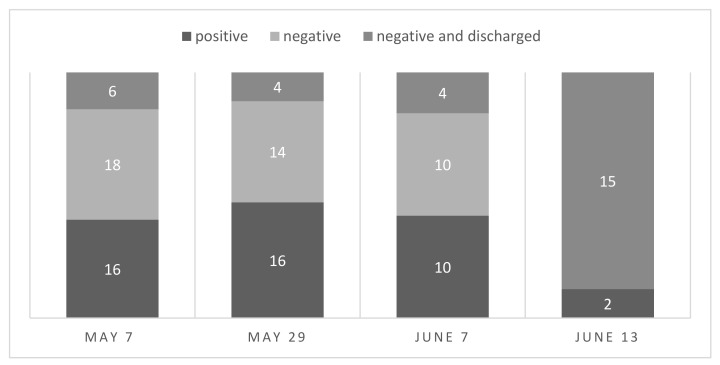
PCR positive and negative results of quarantine populations at different dates.

On 2 May, the date of hospital closure, all febrile horses, six in total, were tested for EHV-1 by PCR on nasopharyngeal swab extracts, with three individuals testing positive for EHV-1. The first series of tests on 7 May were conducted before moving the hospital population to the newly installed quarantine zone, again by PCR on nasopharyngeal swab extracts. All hospitalized horses were tested to identify the EHV-1-positive individuals. At that time, there were eight febrile individuals (of 40 inpatients), who were consequently tested by PCR on venous blood collected in EDTA. One mare tested negative on nasopharyngeal swab but positive on venous blood, and as such was considered positive for EHV-1. In total, there were 16 individuals positive for EHV-1 out of 40 tested. Before the second series of tests, six “negative” horses left the hospital to continue quarantine at home, where they were held in complete isolation from other horses. Two horses from the original hospital population died due to the original gastro-intestinal problems, and two additional horses joined the quarantine population after showing neurological signs on a neighboring premise (C). The consecutive test rounds were by PCR on nasopharyngeal swabs only. The second series of tests on 29 May revealed 16 positive and 18 negative individuals, of which the PCR negative individuals were discharged. The horses that tested negative had previously tested positive, and had been afebrile for over 3 weeks. The third series, one week later on 7 June, revealed 10 positive cases of 24 tested individuals. The last series of samples took place one week later on 13 June, with only two foals testing positive. Figure 2 is an overview of the results of all PCR tests from the quarantine population, associated with results. All horses that tested negative on 13 June were allowed to go home. This means that 10 cases of the 40 individuals needed a second test (28 days after last reported fever) and seven cases of the 40 individuals needed a third test (35 days after last reported fever) before a negative result was obtained. The two remaining foals tested negative two weeks later, on 26 June, which meant they had been shedding the virus for at least 49 days (“super shedders”). One horse who tested negative in the third series of tests on 7 June, and had subsequently gone home, became pyrexic and tested positive again on 26 June. This means that the horse relapsed within 19 days.

In total, six (15%) horses from the hospital quarantine population developed neurological symptoms. Three of them remained 1/5 ataxic, without dysuria. The other three made a full recovery.

### 3.2. Outcome of the Infected Premises

There were four commercial and three private equine properties affected by the outbreak. Table 2 is a presentation of measures taken at every premise linked with the EHV-1 outbreak at WEH. It is shown for every premise how many test rounds were carried out and how long horses had been in quarantine.

### 3.3. Total Costs for the Hospital

We can divide the total cost incurred for the hospital during the outbreak into five categories: staff and public relations, hospital revenue (cases seen) loss, material for quarantine, diagnostic testing incl. medication and stable costs. The costs of the outbreak to the hospital are presented in Table 3. The hospital allocated as many of its services as possible to the ambulatory practice, resulting in extra working hours for the ambulatory team. Cleaning and disinfection were performed by hospital staff. Helpdesk and informative meetings were organized and paid for by the hospital. Setting up the quarantine, and the purchase of new materials, were covered by the hospital. All testing of quarantined horses and the extra treatment costs were covered by the hospital. Sales loss was attributable to the closing of the clinic, transfer of patients to other veterinary practices and negative publicity on social media.

## 4. Discussion

Equine hospitals are challenging environments in which to manage biosecurity, with an intrinsically high biosecurity risk. The goal should always be to protect patients from nosocomial diseases whilst also protecting the integrity and reputation of the hospital [18]. Slater describes three pillars of a biosecurity plan: (i) to control admissions, (ii) contain disease and (iii) clean up after disease. The first pillar represents preventing admitting patients with contagious diseases into the main hospital area. Every hospital needs to have a protocol in place for what to do if a horse enters the hospital or when a hospitalized horse develops fever of unknown origin. In case of WEH, a suspected case of EHV-1 will not be admitted. Horses for elective surgery are admitted the day before the scheduled surgery, so they can undergo a pre-anesthetic exam. The second pillar, containment of the disease, is possible by setting up protocols for personal protection equipment and isolation, with additional hygiene requirements and restrictions on movement of people and horses. Hospitalized horses who develop fever of unknown origin will be transferred to the isolation. Our equine hospital at WEH is divided into three barns and, despite this layout, the fever spread between barns. Slater also discusses the concept of an epidemiological unit, when restrictions need to be applied to groups of horses or even whole sections of a hospital. In the event of suspected contagious respiratory disease and/or contagious neurological disease, restrictions will be unavoidable [19]. This means that in the case of EHV-1-associated EHM, the hospital will have to manage horses that are stabled in different departments as epidemiological groups. However, “distance” between groups is the best method of protection. In our experience, partial quarantine or isolation was impossible to maintain at our equine hospital during the outbreak of EHV-1, with the relocation of the entire infected population being considered as the best alternative solution. This allowed the hospital to reopen after thorough cleaning and disinfection. The third pillar is to prevent onward transmission from the contaminated environment (nosocomial infection), and for this, a hospital needs an evidence-based cleaning and disinfection protocol [18]. Every stable should first be cleaned with water and soap followed by 12 h of drying. Afterwards, the stables should be disinfected with chloride solution or another phenolic-based disinfectant, rinsed with water and left to dry again for 12 h.

The treatment of an EHV-1-infected individual is aimed towards preventing the development of EHM by reducing the pro-inflammatory host response during the early onset of viremia. This therapeutic approach helps prevent the endothelial cells from becoming responsive to the attachment of lymphocytes and subsequently allowing transfer of the virus into the endothelial cells. A study by Goehring et al. (2017) showed that the infection rate of endothelial cells under the influence of anti-inflammatories (firoxocib, flunixin meglumine, lidocaine and dexamethasone) was decreased in vitro [19]. The use of antivirals may decrease the viral load and thereby reduce the chance of developing EHM, however, an experimental study by Garré et al. (2009) was unable to show a decrease in nasal shedding or viremia [20]. A similar study by Maxwell et al. (2017) showed that EHM severity was less compared to an untreated control group; however, only if treatment with antiviral medication (valacyclovir) was started very early on in the course of the disease [21]. This could be beneficial for other horses in the group if they receive valacyclovir as soon as the first horse is diagnosed with EHV-1, and due to the fact that the patent for valacyclovir has expired, this has become a cheaper and more accessible option.

When to lift quarantine restrictions from premises can be a challenging decision, as there are multiple criteria presented by various experts to take into consideration, and no one protocol is 100% secure with regard to further spread of the disease. In the event of a neurological outbreak of EHV-1, it is important to consider all in-contact horses involved to be infectious until at least 21 days after the onset of clinical signs [6]. The recent AAEP guidelines extended this to 28 days because of evidence of more protracted shedding in some clinical cases of EHM [17]. The use of diagnostic testing to determine the status of viral shedding is the most pragmatic approach [18], with PCR testing beginning 14 days after the last day of recorded fever [6]. WEH used this approach, although the discharged horses only tested negative on nasopharyngeal swabs taken at least 21 days after the last day of recorded fever, with the expectation that horses were no longer shedding once testing negative. There was only one horse who developed a fever (19 days later) after a negative test result using this method. This shows that even after going through a natural EHV-1 infection, the possibility of recrudescence of latent virus may occur even after a short period of time. Goehring et al. released patients after a period of 14 days counted from the last day of fever in the last horse with fever, without EHM cases in the interim, and after three consecutive negative tests. In a primary infection in adult horses, the nasal shedding may occur intermittently and only for a short duration of time, giving the possibility of false negative test results [22]. During the outbreak in WEH, 10 cases of the 40 individuals needed second tests (28 days after last reported fever) and seven cases of the 40 individuals needed a third test (35 days after last reported fever) before a negative result was obtained. There were two remaining foals who tested negative 49 days after the last day of reported fever.

It is strongly advised that the occurrence of an outbreak is immediately reported to the responsible authorities; in the Netherlands, this would be the Sectorraad Paarden (SRP), the National Sport Federation (KNHS), the National Animal Health Service (Royal GD), the Veterinary Association (NEVA) and the Faculty of Veterinary Medicine at Utrecht University. A meeting for all hospital staff should be organized, for briefing and discussing the protocol of actions. In order to prevent spread of the disease and inform clients, horse owners and the public, general information and accurate situation reports should be shared via the hospital’s website and social media platforms., as early communication is key in limiting the spread of disease, but also in maintaining the affected premises’ reputation and integrity.

EHV-1 is not a notifiable disease in the Netherlands, and as such there are no regulations for establishing quarantine of a facility where an outbreak of EHV-1 has been reported. A national surveillance system was established in 2019, Surveillance Equine Infectieziekten Nederland (SEIN). SEIN is organized by analogy of the French surveillance system (Réseau d’EpidémioSurveillance des maladies équines Européen, RESPE) and was established by the National Veterinary Association and the National Animal Health Service (Royal GD). The system is designed to swiftly gather and distribute information from veterinarians with regard to infectious diseases confirmed by appropriate tests. The parties involved aim to provide up-to-date information via SEIN for all parties interested in reliable health information that can be used in the fight against epidemics. WEH decided to include, in their hospital protocol, a signed document on admission in which both parties agree that horses can be tested at any time when suspected of EHV-1 and that results will be shared with SEIN. Also, that no costs and liability will be covered by WEH.

The potential risk of unknowingly admitting an EHV-1-infected horse into a hospital is real, and should be considered in light of the financial repercussions that can ensue. The primary source of the EHV-1 infection in the WEH outbreak was never definitively identified, although multiple scenarios were considered possible. Most likely, regarding the chronologic order of events, the horse of Premise A had started shedding whilst at the hospital. This horse underwent orthopedic surgery with a significant amount of stress and pain. The horse of Premise D (for insemination with frozen semen) was housed in the same stable barn. The hospitalization of patients shedding EHV is a serious risk to maintaining proper biosecurity in equine hospitals. At WEH, biosecurity is maintained following the guidelines as described by Slater [19]. However, even then, the hospital is required to close the premises according to the current guidelines. This risk may increase in future due to the increased movement of competition and breeding horses. Hospitals are not insured to cover the expenses incurred and at the same time are expected to manage an outbreak following the nationally issued guidelines. The current outbreak occurred at the beginning of May which is the busiest period of the year and, with that, a huge loss of income when comparing revenue to previous years. In order to avoid legal claims and billing issues, the hospital paid for all patient care directly related to EHV-1. With this, we managed to succeed in maintaining satisfied clients, complying with all the measures required with their horses. However, 3 months later, a yearling was brought in because of a rectum prolapse. It developed fever and tested positive on nasopharyngeal swab for EHV-1. It was immediately confined to quarantine following hospital biosecurity regulations. Even though EHV-1 had already been circulating in this trading stable, all costs for loss of revenue, deceased horses, treatment and testing of other horses at that stable were imposed as a liability claim on WEH (EUR 120,000). Fortunately, this claim was dismissed based on the pronunciation of an independent expert bureau hired by the insurance company.

As with most infectious diseases, prevention is the best option, and therefore a well-thought-out vaccination program in combination with sensible disease monitoring and biosecurity measures is key in limiting the spread of disease. Vaccination against EHV-1 is not compulsory in sports federations in the Netherlands, with the only exception of the Dutch Standardbred Racing Association (Nederlandse Draf en Rensport—NDR), by analogy of the French Horse Racing Federation (Federation Nationale des Courses Hippique). Pre-exposure prophylaxis through vaccination would result in fewer horses becoming infected during contact with shedding horses, whilst also being less likely to develop viremia and EHM [23]. Vaccination, however, can only be a successful tool in disease prevention if a large percentage of horses (>80%) are vaccinated. 

The Equine Herpesvirus-1 Consensus Statement ACVIM (2009) noted that there is a lack of evidence that current vaccines prevent naturally occurring cases of EHM. However, the control of cell-associated viremia is important, and vaccination has been shown to reduce or eliminate cell-associated viremia in a controlled experiment and to stimulate the immune response [17]. A study by Goehring et al. (2010) showed that EHV-1 vaccination is an aid in reducing clinical signs of infection and viremia. The reduction in clinical symptoms, including pyrexia, was significantly greater with modified live vaccines than with inactivated vaccines [23]; however, a significant decrease in viremia was noticed in the group of vaccinates with an inactivated vaccine. Goodman et al. (2006) concluded in their study that a modified vaccine did offer better protection against EHM when compared with polyvalent vaccines (including the EHV-1 antigen) [24]. The highest level of immunity occurs shortly after vaccination, and there is then a steady decline in active immunity components, such as antibodies and cytotoxic lymphocytes, alongside a steady decline in memory. With revaccination (or a “booster”), a swift boost in immunity is to be expected, once again followed by a steady decline. The protection offered by a vaccine depends on the severity of the challenge and the interval between vaccination and challenge [23]. In 2003, Patel et al. performed a study where a modified live EHV-1 vaccine was administered intranasally, and subsequently protected approximately 80% of mares from aborting when each one was experimentally challenged, also intranasally. None of the vaccinated mares shed the virus or became viremic following a challenge with the infectious agent [25]. With this in mind, a similar vaccine may possibly be developed to protect horses from neurological disease caused by EHV-1, similar to the intranasal modified live vaccine for infectious bovine rhinotracheitis (IBR) in cattle.

## Figures and Tables

**Table 1:** List of possible case 1 source.

Premises	Horse	Reason for Admission	Days in Hospital	Number of Days Discharged before Hospital Closure
A	Warmblood, mare	lameness ex	2 days	15 days
B	Warmblood, male	castration	3 days	6 days
C	Quarter horse, mare	insemination	3 days	3 days
D	Quarter horse, mare	insemination	8 days	12 days
E	Friesian mare and foal	patent urachus (foal)	10 days	1 day
F	Warmblood, mare	insemination	2 days	1 day
G	Friesian, mare	fever	3 days	7 days

**Table 2:** Overview of the infected premises by the outbreak.

Premises	Horses Tested Positive (PCR) with or without Symptoms	Test Rounds at Premises and (PCR) Results	Total Days of Isolation
A	1 with neurological symptoms Positive test	2 test rounds All negative	25 days
B	1 with fever Positive test	2 test rounds All negative	28 days
C	1 with fever Positive test	1 test round All negative	18 days
D	1 with mild neurological symptoms Positive test	Multiple test rounds until all horses tested negative. With the first test round, 6 tested positive	30 days
E	1 foal with fever Positive test	No test rounds; 2 foals and 2 mares became febrile	30 days Until every horse was afebrile
F	1 with fever No testing	No test rounds	None
G	1 with fever No testing	No test rounds	Only 1 mare with fever isolated for 21 days

**Table 3:** All costs incurred by the hospital, divided into five categories, presented in euros (€).

Category	Costs (Euros)
Staff and public relations	10,806
Loss of revenue	76,805
Materials for quarantine	26,887
Stabling costs	31,896
Medication	4606
**Total**	**151,000**

## Data Availability

Not applicable.
